# Utilisation of exome sequencing for muscular disorders in Thai paediatric patients: diagnostic yield and mutational spectrum

**DOI:** 10.1038/s41598-023-28405-6

**Published:** 2023-01-25

**Authors:** Sarinya Summa, Chupong Ittiwut, Pimchanok Kulsirichawaroj, Tanitnun Paprad, Surachai Likasitwattanakul, Oranee Sanmaneechai, Ponghatai Boonsimma, Kanya Suphapeetiporn, Vorasuk Shotelersuk

**Affiliations:** 1 Department of Paediatrics, Faculty of Medicine Siriraj Hospital, Mahidol University, Bangkok, 10700 Thailand; 2 Department of Paediatrics, Samutprakan Hospital, Samutprakan, 10270 Thailand; 3 Center of Excellence for Medical Genomics, Medical Genomics Cluster, Department of Paediatrics, Faculty of Medicine, Chulalongkorn University, Bangkok, 10330 Thailand; 4 Excellence Center for Genomics and Precision Medicine, King Chulalongkorn Memorial Hospital, the Thai Red Cross Society, Bangkok, 10330 Thailand; 5 Department of Paediatrics, Faculty of Medicine Vajira Hospital, Navamindradhiraj University, Bangkok, 10300 Thailand; 6 Division of Neurology, Department of Paediatrics, Faculty of Medicine, Chulalongkorn University, Bangkok, 10330 Thailand

**Keywords:** Genetic testing, Neuromuscular disease, Next-generation sequencing

## Abstract

Muscular dystrophies and congenital myopathies are heterogeneous groups of inherited muscular disorders. An accurate diagnosis is challenging due to their complex clinical presentations and genetic heterogeneity. This study aimed to determine the utilisation of exome sequencing (ES) for Thai paediatric patients with muscular disorders. Of 176 paediatric patients suspected of genetic/inherited myopathies, 133 patients received a molecular diagnosis after performing conventional investigations, single gene testing, and gene panels. The remaining 43 patients from 42 families could be classified into three groups: Group 1, MLPA-negative Duchenne muscular dystrophy (DMD) with 9 patients (9/43; 21%), Group 2, other muscular dystrophies (MD) with 18 patients (18/43; 42%) and Group 3, congenital myopathies (CM) with 16 patients (16/43; 37%). All underwent exome sequencing which could identify pathogenic variants in 8/9 (89%), 14/18 (78%), and 8/16 (50%), for each Group, respectively. Overall, the diagnostic yield of ES was 70% (30/43) and 36 pathogenic/likely pathogenic variants in 14 genes were identified. 18 variants have never been previously reported. Molecular diagnoses provided by ES changed management in 22/30 (73%) of the patients. Our study demonstrates the clinical utility and implications of ES in inherited myopathies.

## Introduction

Muscular dystrophy (MD) and congenital myopathy (CM) are heterogeneous groups of inherited muscular disorders that result in significant disability in children and adults. MD is characterised by progressive muscle weakness and dystrophic pathological features on muscle biopsy. MD has been conventionally classified according to age at onset, main clinical and biopsy findings, and immunohistochemical staining. Examples of MD are dystrophinopathies, sarcoglycanopathies and dysferinopathies. However, many of the defective proteins involved in MD cannot be readily determined.

CM is a group of genetic muscle disorders characterised clinically by hypotonia and weakness, usually from birth, and a static or slowly progressive clinical course. Historically, the classification of CM has been based on the major morphological features seen on muscle biopsy.

Due to substantial advances in molecular genetics over the last 2 decades, neuromuscular disorders can now be diagnosed through molecular genetic testing^1,2^. More than 100 genes related to neuromuscular disorders have been identified to date^3^.

In paediatric patients, the clinical features of neuromuscular disorders are mostly nonspecific (such as motor developmental delay, hypotonia and weakness) and rarely adequately characteristic for a definite diagnosis. Next-generation sequencing (NGS) is increasingly used for molecular diagnosis in neuromuscular diseases. By combining the phenotype of a patient, pathological findings from muscle biopsy and sequencing data, causative genes can now be efficiently identified^4,5^.

We sought to evaluate the diagnostic yield and clinical utility of exome sequencing (ES) in paediatric patients with CM and MD at 2 tertiary-care centres in Thailand.

## Materials and Methods

### Study design and patient population

**Figure 1:**
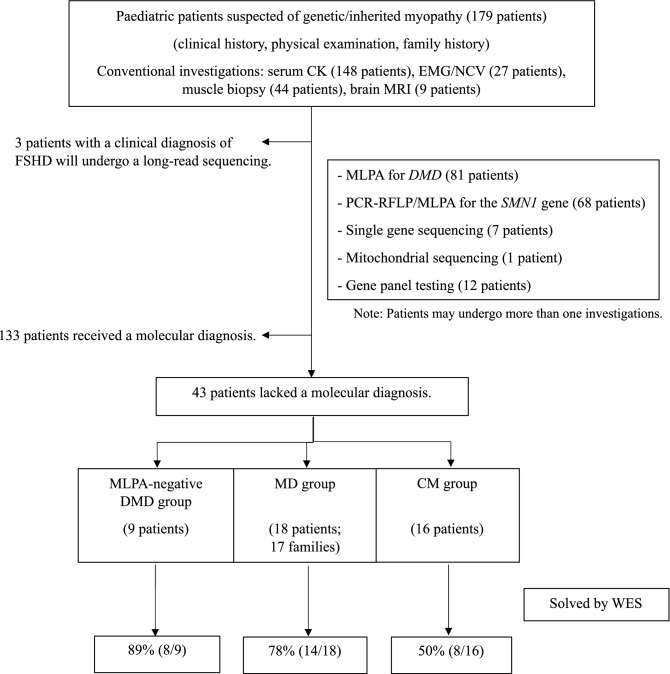
Patient enrolment workflow.

This research was conducted in accordance with the Declaration of Helsinki. The descriptive, cross-sectional study was performed on paediatric patients (age ≤ 18 years) with MD and CM from Siriraj Neuromuscular Disease Center (Siriraj Hospital) and King Chulalongkorn Memorial Hospital in Thailand between September 2018 and April 2021. The diagnoses of MD and CM were made by paediatric neurologists and based on the presence of muscle weakness, age at onset, clinical course, physical findings, conventional investigations and muscle histopathological findings. Muscle biopsies were performed using standard immunocytochemistry protocols. Staining for dystrophin, utrophin, merosin, dysferlin, caveolin-3, dystroglycan, sarcoglycan, emerin, collagen VI and desmin was performed. Single-gene molecular tests were performed on patients with suggestive clinical and pathological findings. The molecular tests were multiplex ligation-dependent probe amplification (MLPA) for spinal muscular atrophy (*SMN1*) and DMD (*DMD*); Sanger sequencing of coding regions of *RYR1* (8 out of 106 exons) and *ACTA1*; and mitochondrial DNA sequencing (A3243G, A8344G, T8993G). Patients suspected of having facioscapulohumeral MD (FSHD) underwent long-read DNA sequencing. Patients whose mutations were still elusive were subjected to ES (Fig. 1).

### Ethic approval statement

Before this research began, its protocol was approved by the Siriraj Institutional Review Board (protocol number 343/2560 (EC4) and approval number 264/62).

### DNA extraction, exome sequencing and bioinformatic analysis

Informed consent was obtained from the subjects or their parents to collect 3–5 millilitres of the participants’ peripheral blood. Genomic DNA was isolated from leukocytes and prepared as an Illumina sequencing library enriched by the SureSelect Human All Exon V5 Kit. The captured libraries were sequenced using Illumina HiSeq 4000. The following criteria were used to filter SNVs and Indels: (1) located in exons or flanking introns, (2) nonsynonymous, (3) rare with 1000G minor allele frequency of less than 1%, (4) less than 0.1% in the Genome Aggregation Database (gnomAD), (5) less than 10 alleles in Thai reference exome (T-Rex)^20^ and 2166 in-house Thai exome controls, (6) (if the variant is a missense) predicted to be damaging by SIFT and Polyphen, or (7) related to the phenotype of the patient.

The variants in genes known to cause neuromuscular diseases were first analysed^3^. All candidate variants were evaluated by clinical geneticists and neurologists, who classified them according to the American College of Medical Genetics and Genomics interpretation guidelines^21^. Variants were considered ‘novel’ if they had not been reported in Pubmed (https://pubmed.ncbi.nlm.nih.gov/).

Cases were considered ‘solved’ if they had pathogenic or likely pathogenic variants in genes consistent with the phenotype and if their zygosity test results matched the inheritance pattern. However, cases were considered ‘partially solved’ if only 1 variant in a gene was associated with recessive inheritance of a disorder.

## Results

Forty-three patients from 42 unrelated families underwent ES. Sixty-three percent (27/43) were male. The age at disease onset ranged from birth to 10 years, with a median age at onset of 18 months (Table 1). Nine patients (21%) were diagnosed with negative-MLPA DMD, 18 (42%) with other types of MD, and 16 (37%) with CM (Fig. 1). Muscle biopsies were performed on 44% (4/9) of the DMD group, 78% (14/18) of the MD group and 50% (8/16) of the CM group; the overall rate was 60% (26/43). Trio-ES was performed on 33 patients from 32 families (77%), while singleton-ES was performed on 10 patients (23%).

**Table 1:** Demographic data.

	Frequency
Sex, N (%)	
Male	27 (62.8%)
Female	16 (37.2%)
Age, median (range)	
Current age	8Y2M (0–25Y)
Age of onset	18M (0–10Y)
Age at clinical diagnosis	7Y4M (0–25Y)
Clinical diagnosis, N (%)	
Congenital myopathy	16 (37.2%)
Muscular dystrophy	27 (62.8%)

**Figure 2:**
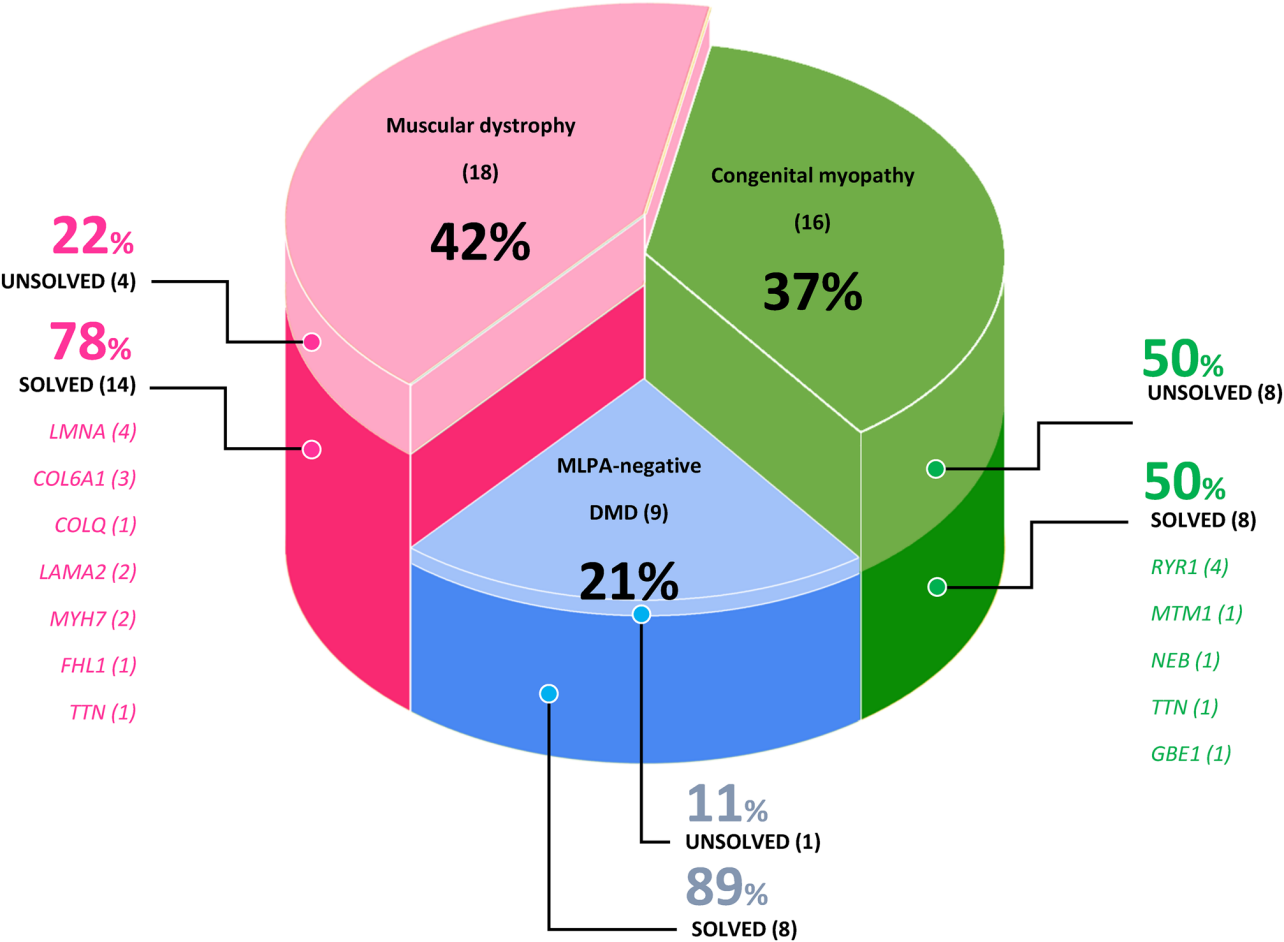
Molecular diagnoses by disease group. Molecular diagnoses using exome sequencing were established in 70% of the patients. The number of patients is given in parentheses.

Disease-causing variants were identified in 30 patients (70%; 30/43). Twenty-eight cases were solved, leaving only 2 cases that were partially solved (see supplementary Table S1 online). Thirty-six pathogenic/likely pathogenic variants were found in 14 genes (Fig. 2). The clinical characteristics of the patients and the disease-causing genes are summarized in the supplementary Table S1. The molecular findings are summarized in supplementary Table S2. Eighteen novel variants were identified (supplementary Table S3 online). Thirteen novel variants were classified as pathogenic or likely pathogenic, and 5 variants were classified as variants of unknown significance (VUS).

### The MLPA-negative DMD group

ES identified the responsible variants in 8 of the 9 patients in the MLPA-negative DMD group. Each variant was different. All 8 were point mutations (5 frameshifts, 1 splice-site, 1 nonsense and 1 inframe deletion) that had not been identified by MLPA theoretically or in practice. Two frameshift variants had never been reported (supplementary Table S3 online). Interestingly, the nonsense variant, c.10108C>T (p.Arg3370Ter), in Patient DMD8 was a candidate for premature stop codon read-through treatment (Ataluren)^6^.

### The MD group

Among the 18 patients with other types of MD, disease-causing variants were identified in 14 (78%). Twelve of those 14 cases were solved, and the other 2 were partially solved. Fifteen pathogenic and likely pathogenic variants were identified in 7 genes (see supplementary Table S2 online). Four of the variants had never been reported. The modes of inheritance of these cases were autosomal dominant in 64% (9/14 patients; confirmed de novo 7; inherited 1; not confirmed 1), autosomal recessive in 29% (4/14 patients) and X-linked in 7% (1/14 patients). The causative genes for MD were *LMNA* (n = 4), *COL6A1* (n = 3), *LAMA2* (n = 2), *MYH7* (n = 2), *TTN* (n = 1) and *FHL1* (n = 1). The 2 partially solved cases were Patients MD6 and MD7; both were clinically diagnosed with merosin-deficient MD.

ES led to a diagnosis other than MD in Patient MD4, who presented with congenital hypotonia with weakness, recurrent pneumonia, ptosis and ophthalmoplegia. A muscle biopsy showed faint alpha-dystroglycan and delta-sarcoglycan, leading to the clinical suspicion of alpha-dystroglycan or delta-sarcoglycan deficient MD. Repetitive nerve stimulation did not reveal electrodecremental changes. Surprisingly, ES identified a homozygous splice-site c.393+1G>A variant in *COLQ*, which is known to cause congenital myasthenic syndrome (OMIM#603034).

In Patient MD15, a likely pathogenic variant was found in a gene beyond the scope of clinical presentations. He presented with progressive proximal muscle weakness, areflexia, elevated serum CPK and a deceased male sibling with similar clinical findings (see supplementary Table S1 online). A homozygous missense c.616G>A (Gly206Ser) variant in the *FUS* gene, known to cause familial amyotrophic lateral sclerosis, was found.

### The CM group

ES provided genetic diagnoses in 8 (50%) of 16 patients with CM. Of the 8 cases, 6 (75%) were inherited in an autosomal recessive manner, 1 (12.5%) was de novo, and 1 (12.5%) was X-linked. Fourteen causative variants were identified in 5 genes: *RYR1* (n = 4), *NEB* (n = 1), *MTM1* (n = 1), *TTN* (n = 1), *GBE1* (n = 1). Ten variants had never been reported.

## Discussion

We performed ES in 43 Thai paediatric patients with MD or CM from 42 unrelated families. Their clinical features and WES data were analysed simultaneously, enabling the correlation of the ES results with other diagnostic modalities. The overall diagnostic yield for ES was 70% (30/43). The most common MD-disease genes were *DMD* (30%; 8/27), *LMNA* (15%; 4/27) and *COL6A1* (11%; 3/27), while the most prevalent CM-disease gene was *RYR1*. These gene profiles are like those reported by other studies in paediatric populations^7,8^.

Earlier research on ES in paediatric neuromuscular patients produced diagnostic yields ranging from 37 to 65%^8–11^. The high diagnostic rate for ES in the current investigation (70%) was probably due to several reasons. The patients referred to the 2 tertiary-care centres in this study were highly selective and strongly suspected to have genetic disorders. Most of the patients in the cohort had infantile-onset weakness. This study also included the MLPA-negative DMD subgroup, in which the diagnostic rate was high. In addition, ES was performed as an early diagnostic step. Unlike the practice of many other countries, most of the patients in this cohort did not first undergo extensive serial gene or targeted panel testing. Consequently, ES would have shown a higher level of positive results than otherwise, making ES a contributing factor to the diagnostic yield.

A limitation of using ES early is that it cannot detect molecular pathologies in congenital myotonic dystrophy type 1 (repeat expansion in the *DMPK* gene) and FSHD (contraction of D4Z4 repeats on chromosome 4q35). The 2 disorders are the most common hereditary myopathies after DMD in adult, accounting for 10–35% and 6–10% of hereditary myopathies, respectively^12,13^. This study excluded patients with clinically suspected FSHD without molecular confirmation. Although none of the patients in our cohort had phenotypes specific to congenital myotonic dystrophy type I, infantile and childhood-onset congenital forms can be indistinguishable from other myopathies. By excluding congenital myotonic dystrophy type I and FSHD, mutation-targeted testing could increase the apparent diagnostic yield of ES.

ES changed the management of 73% (22/30) of the diagnosed cases (see supplementary Table S1 online). Muscle biopsies were unnecessary once the molecular diagnosis was made. The confirmation of DMD point mutations allowed for steroid treatment, while identifying the nonsense mutation made Patient DMD8 a candidate for premature stop codon read-through treatment (Ataluren)^6^. The other patient in which ES unravelled the diagnosis and allowed for choice of medication was the patient with *COLQ*-congenital myasthenic syndrome^14^. The findings of this study highlight the utility of ES in ending diagnostic odyssey and permitting precision treatment.

In the MPLA-negative DMD group, the vast majority of cases were solved. One patient in the MLPA-negative DMD group who had clinical findings and muscle histopathology consistent with dystrophinopathy but with no variant found was believed to have a regulatory/promoter or deep intronic alteration. In previous studies on patients whose clinical and histopathological data suggested DMD, ES led to alternative diagnoses of inherited MD^15^. We found no disease-causing variants in other genes. In the future, targeted NGS could be used for patients with MLPA-negative DMD in our setting.

Although our three patients with *COL6A1*-associated muscular disorders (Patients MD1-MD3) were found to harbor only one variant, there is a possibility that they are actually compound heterozygous with the other variant not identified by our methods.

Patients MD6 and MD7 had delayed motor milestones, proximal muscle weakness, and diffuse white matter hypersignal intensity on brain MRI. Muscle biopsies showed faint and absent merosin staining in Patients MD6 and MD7, respectively. The clinical diagnosis was autosomal recessive merosin-deficient congenital MD (OMIM#607855)^16^. In Patient MD6, trio-ES identified a previously reported heterozygous splice-site c.283+1G>C variant in *LAMA2*. In Patient MD7, singleton-ES identified a novel heterozygous frameshift variant, c.2718delT (p.Phe906LeufsTer169). The patient’s clinical, histopathological and ES data led to a diagnosis of autosomal recessive merosin-deficient congenital MD. This diagnosis suggested that a second variant in trans was missed in each case. Mutations that have structural variants or variants in noncoding regions cannot be identified by ES. Both patients will undergo long-read genome sequencing.

The phenotypes of *TTN*-related myopathy in this cohort were diverse. *TTN* variants were found in both the CM and MD groups. Patient MD14, categorised into the MD group, had arthrogryposis multiplex congenita, finger/elbow flexion contractures and proximal muscle weakness. A muscle biopsy showed nonspecific myopathic changes and faint collagen VI staining. The clinical characteristics of Patient MD14 mimicked collagenopathy. Patient CM9 had perinatal onset hypotonia with weakness, respiratory failure and cardiomyopathy, and a muscle biopsy revealed centrally located nuclei and fibre-type disproportion. Neither Patient MD14 nor Patient CM9 had ptosis or ophthalmoplegia. ES identified *TTN* compound heterozygous truncating and missense variants in both patients. Recent evidence has shown that missense mutations associated with the disease exert their effects when they occur with a truncating mutation. Previously reported cases with *TTN* compound heterozygous truncating and missense variants typically presented at birth. Their clinical courses were characterised by variable progression of weakness, contractures, scoliosis and respiratory symptoms but spared the extraocular muscles^17^.

A likely pathogenic variant was found in a gene causing the phenotype beyond the clinical features described in this study. Patient MD15 presented with progressive muscle weakness, areflexia and elevated serum CPK with a family history of a deceased male sibling with similar clinical findings. Muscle biopsies from Patient MD15 and his male sibling showed vacuolated muscle fibres with granular eosinophilic and amphophilic materials, cyclooxygenase (COX)-negative fibres and numerous abnormal mitochondria on electron microscopy. These findings suggested mitochondrial defects. Causative variants were not identified in nuclear- or mitochondrial-related genes. Trio-ES analysis revealed a homozygous missense c.616G>A (Gly206Ser) variant in the *FUS* gene. This variant has been reported in a heterozygous state in adult patients with familial autosomal dominant amyotrophic lateral sclerosis (OMIM#608030)^18^.

To our knowledge, there has been only 1 report of a homozygous *FUS* variant in a family with adult-onset amyotrophic lateral sclerosis with an autosomal recessive pattern of inheritance^19^. The missense c.616G>A (Gly206Ser) variant is rare, with a gnomAD allele frequency of 0.0000649. In addition, no homozygotes were found in the healthy population database (https://gnomad.broadinstitute.org/). The clinical presentation of Patient MD15 did not support a diagnosis of motor neuron disease. Therefore, the c.616G>A (Gly206Ser) variant in *FUS* is classified as likely pathogenic but not playing an etiological role in Patient MD15’s phenotype. The clinical significance of discovering this variant would be to provide appropriate genetic counselling about the risk of a currently untreatable disease for asymptomatic parents in their 30 s.

Increasing evidence shows that NGS technology, including targeted NGS and ES, has high clinical utility and saves time and costs^7^. However, there are presently no universally accepted guidelines for genetic testing in paediatric neuromuscular patients. In our tertiary-care setting, we employed ES since the clinical presentation in most patients did not point to a specific disease except in the MLPA-negative DMD group. In the DMD group, targeted NGS may be an option in the future. With other patients, limited gene testing carries the risk of missing disorders beyond provisional diagnosis. The cost-effectiveness of using ES early in our setting remains to be explored.

## Conclusions

This study shows the efficiency of ES in providing disease-causing variants in Thai paediatric patients clinically diagnosed with CM and MD. ES can also unravel the diagnosis of rare diseases presenting with muscle weakness and overlapping phenotypes with CM and MD. In turn, this leads to precision therapy and appropriate genetic counselling.

## Supplementary Information

Supplementary Tables.

## Data Availability

Data of 18 novel mutations are publicly available from ClinVar database: https://www.ncbi.nlm.nih.gov/clinvar/submitters/507386/. The ClinVar accession numbers for this submission are SCV002546536-SCV002546549 and SCV002546551-SCV002546554.

## References

[1] Mercuri E, Muntoni F (2013). Muscular dystrophies. Lancet.

[2] North KN (2014). Approach to the diagnosis of congenital myopathies. Neuromuscul. Disord..

[3] Benarroch L, Bonne G, Rivier F, Hamroun D (2020). The 2021 version of the gene table of neuromuscular disorders (nuclear genome). Neuromuscul. Disord..

[4] Valencia CA (2013). Comprehensive mutation analysis for congenital muscular dystrophy: A clinical PCR-based enrichment and next-generation sequencing panel. PLoS ONE.

[5] Bohm J (2013). An integrated diagnosis strategy for congenital myopathies. PLoS ONE.

[6] McDonald CM (2017). Ataluren in patients with nonsense mutation Duchenne muscular dystrophy (ACT DMD): A multicentre, randomised, double-blind, placebo-controlled, phase 3 trial. Lancet.

[7] Chae JH (2015). Utility of next generation sequencing in genetic diagnosis of early onset neuromuscular disorders. J. Med. Genet..

[8] Harris E (2017). Exome sequences versus sequential gene testing in the UK highly specialised Service for Limb Girdle Muscular Dystrophy. Orphanet. J. Rare Dis..

[9] Waldrop MA (2019). Diagnostic utility of whole exome sequencing in the neuromuscular clinic. Neuropediatrics.

[10] Todd EJ (2015). Next generation sequencing in a large cohort of patients presenting with neuromuscular disease before or at birth. Orphanet. J. Rare Dis..

[11] Babic-Bozovic I (2021). Diagnostic yield of exome sequencing in myopathies: Experience of a Slovenian tertiary centre. PLoS ONE.

[12] Cotta A (2017). The relative frequency of common neuromuscular diagnoses in a reference center. Arq. Neuropsiquiatr..

[13] Pagola-Lorz I (2019). Epidemiological study and genetic characterization of inherited muscle diseases in a northern Spanish region. Orphanet. J. Rare Dis..

[14] Finsterer J (2019). Congenital myasthenic syndromes. Orphanet. J. Rare Dis..

[15] Luce LN (2018). Small mutation screening in the DMD gene by whole exome sequencing of an argentine Duchenne/Becker muscular dystrophies cohort. Neuromuscul. Disord..

[16] Oliveira, J., Parente Freixo, J., Santos, M. & Coelho, T. In *GeneReviews((R))* (eds Adam, M. P. *et al.*) (1993).

[17] Rees M (2021). Making sense of missense variants in TTN-related congenital myopathies. Acta Neuropathol..

[18] Yan J (2010). Frameshift and novel mutations in FUS in familial amyotrophic lateral sclerosis and ALS/dementia. Neurology.

[19] Kwiatkowski TJ (2009). Mutations in the FUS/TLS gene on chromosome 16 cause familial amyotrophic lateral sclerosis. Science.

[20] Shotelersuk V (2021). The Thai reference exome (T-REx) variant database. Clin. Genet..

[21] Richards S (2015). Standards and guidelines for the interpretation of sequence variants: A joint consensus recommendation of the American College of Medical Genetics and Genomics and the Association for Molecular Pathology. Genet. Med..

