# Supplementary material for: Utilisation of exome sequencing for muscular disorders in Thai paediatric patients: diagnostic yield and mutational spectrum
Source: Sci Rep. 2023 Jan 25;13:1376. doi: 10.1038/s41598-023-28405-6 (PMC9876991; doi:10.1038/s41598-023-28405-6)
Supplement: Supplementary file 1 — Supplementary Tables. [file 41598_2023_28405_MOESM1_ESM.pdf]

Table S1. Clinical characteristics of the patients and disease-associated genes

**Patient ID**	**Sex**	**Age of onset**	**Clinical feature**	**Disease gene (phenotype /MIM number)**	**Mode of inheritance/ Family history**	**Maximum motor ability**	**Current motor ability**	**Spine deformity**	**Respiratory**[a]	**Cardiac**[a]	**CK (U/l)**	**Edx**	**Muscle biopsy**	**Prior negative genetic testing**	**Management change**	
															**Investigation /surveillance change**	**Treatment change**
**DMD1**	M	1-5Y	Difficulty in standing up, proximal muscle weakness, calf muscle pseudohypertrophy	***DMD*** (Duchenne muscular dystrophy/ 310200)	XR /Negative	Independent walking	Independent walking	Lordosis	Mild restrictive lung disease, snoring with ATH	No	18.930	NE	NE	DMD[b]	Muscle biopsy is not necessary.	Steroid
**DMD2**	M	1-5Y	Proximal muscle weakness	***DMD*** (Duchenne muscular dystrophy/ 310200)	XR /Negative	Independent walking	Wheelchair (loss of ambulation at age 6Y)	No	No	No	9.195	NE	NE	DMD[b]	Muscle biopsy is not necessary.	Steroid
**DMD3**	M	1-5Y	GDD, proximal muscle weakness, calf muscle pseudohypertrophy	***DMD*** (Duchenne muscular dystrophy/ 310200)	XR /maternal uncle with muscle weakness and death during childhood	Independent walking	Independent walking	Scoliosis	No	No	16.836	NE	Dystrophic change, absence of dystrophin staining	DMD[b]	None	None
**DMD4**	M	1-5Y	GDD and proximal muscle weakness, calf muscle pseudohypertrophy	***DMD*** (Duchenne muscular dystrophy/ 310200)	XR /Negative	Independent walking	Independent walking	No	No	No	40.000	NE	NE	DMD[b]	Muscle biopsy is not necessary.	Steroid
**DMD5**	M	1-5Y	Frequent falling, proximal muscle weakness, calf muscle pseudohypertrophy	***DMD*** (Duchenne muscular dystrophy/ 310200)	XR /Negative	Independent walking	Independent walking	Lordosis	No	No	7.708	NE	NE	DMD[b]	Muscle biopsy is not necessary.	Steroid
**DMD6**	M	1-5Y	GDD and proximal muscle weakness	***DMD*** (Duchenne muscular dystrophy/ 310200)	XR /Negative	Independent walking	Wheelchair (loss of ambulation at age 6Y)	No	OSA	No	7.129	NE	Dystrophic change, absence of dystrophin staining	DMD[b]	None	None
**DMD7**	M	1-5Y	Proximal muscle weakness, calf muscle pseudohypertrophy	***DMD*** (Duchenne muscular dystrophy/ 310200)	XR /Negative	Independent walking	Wheelchair (loss of ambulation at age 12Y)	Scoliosis	OSA, NIV need from age 14	LVNC	5.848	NE	End stage muscle disease, dystrophin staining positive	DMD[b]	None	Steroid
**DMD8**	M	1-5Y	Walking difficulty, ADHD, autistic, GDD, epilepsy, calf muscle pseudohypertrophy	***DMD*** (Duchenne muscular dystrophy/ 310200)	XR /Negative	Independent walking	Independent walking	Lordosis	No	No	1.0247	NE	NE	DMD[b]	Muscle biopsy is not necessary.	Candidate for Atarulen treatment

**MD1**	F	1-5Y	Proximal muscle weakness, proximal joint contracture, distal joint hyperlaxity, keratosis pilaris, keloids	***COL6A1*** (Ullrich congenital muscular dystrophy /254090)	AD /Negative	Independent walking	Wheelchair	Rigid spine	OSA, moderate restrictive lung disease, NIV need from age 14	No EF 69%	623	NE	Myopathic change, SSCD	SMA[c]	Surveillance for respiratory involvement.	None
**MD2**	M	1-12M	Severe scoliosis, hip dislocation, hypotonia, proximal muscle weakness, distal joint laxity	***COL6A1*** (Ullrich congenital muscular dystrophy /254090)	AD /Negative	Sitting with aid	Sitting with aid	Scoliosis	No	No EF 70%	300	NE	Myopathic change, SSCD		Surveillance for respiratory involvement	None
**MD3**	M	1-5Y	Proximal muscle weakness, proximal joint contracture, Bethlem sign, distal joint laxity, keratosis pilaris, keloid	***COL6A1*** (Bethlem myopathy /158810)	AD /Negative	Independent walking	Independent walking at the age of 19	Scoliosis	Mild Restrictive lung disease	No	815	Myopathic	Myopathic change		Surveillance for respiratory involvement	None
**MD4**	F	0-1M	Hypotonia recurrent pneumonia, ptosis with ophthalmoplegia	***COLQ*** (congenital myasthenic syndrome/ 603034)	AR /Negative	Sitting alone	Sitting alone	No	OSA	No	50	Repetitive nerve stimulation : normal	Faint alpha-dystroglycan, delta-sarcoglycan		None	Start ephedrine
**MD5**	M	6-10Y	Progressive proximal muscle weakness, elbow joint contracture, hip joint flexion contracture, wing scapular, rigid spine	***FHL1*** (X-linked myopathy with postural muscle atrophy/ 300696)	XR /maternal uncle with muscle weakness since age 10Y	Independent walking	Independent walking	Scoliosis, Rigid spine	No	No	1.931	Myopathic	Myofibrillar change, desmin aggregation	DMD[b]	Surveillance for cardiac involvement	None
**MD6**	M	1-12M	GDD, hypotonia with muscle weakness, diffuse abnormal hypersignal of white matter on brain MRI	***LAMA2*** (congenital, merosin deficient muscular dystrophy/ 607855)	AR /Negative	Sitting alone	Sitting alone	Scoliosis	No	No	3.326	Myopathic	Dystrophic change, faint merosin staining	SMA[c]	None	None
**MD7**	M	1-12M	Delayed motor development, hypotonia with muscle weakness, diffused abnormal hypersignal of white matter on brain MRI, focal epilepsy	***LAMA2*** (congenital, merosin deficient muscular dystrophy/ 607855)	AR /Negative	Sitting with aid	Sitting with aid	No	OSA	No EF 66%	1.432	NE	Dystrophic change, absence of merosin staining	SMA[c]	None	None
**MD8**	F	1-12M	Neck weakness, proximal muscle weakness	***LMNA*** (Emery-Dreifuss muscular dystrophy/ 181350)	AD /Negative	Sit with support, stand holding on	Sit with support, stand holding on	Lordosis	No	No	990	NE	NE	SMA[c]	Muscle biopsy is not necessary. Surveillance for cardiac involvement	None
**MD9**	F	1-5Y	Toe walking, hypertrophic scar at left knee, gait: lordosis	***LMNA*** (Emery-Dreifuss muscular dystrophy/ 181350)	AD /Negative	Independent walking	Dependent walking	Rigid spine, scoliosis, lumbar lordosis	No	NE	194	NE	NE	SMA[c]	Muscle biopsy is not necessary. Surveillance for cardiac involvement	None

**MD10**	F	1-12M	Delayed motor development, proximal muscle weakness, multiple joint contractures	***LMNA*** (Emery-Dreifuss muscular dystrophy/ 181350)	AD /Negative	Sitting alone	Sitting with aid	Scoliosis	OSA, NIV need from age 6	Atrial fibrillation	756	NE	Dystrophic change	SMA[c]	Surveillance for cardiac involvement	None
**MD11**	F	1-12M	Delayed motor development, proximal muscle weakness, both ankle contracture, atrophy of quadriceps muscle	***LMNA*** (Emery-Dreifuss muscular dystrophy/ 181350)	AD /Negative	Independent walking	Independent walking	Lumbar lordosis	No	No	3.266	NE	Dystrophic change	SMA[c]	Surveillance for cardiac involvement	None
**MD12**[e]	F	1-5Y	Frequent falls, slow progressive distal muscle weakness of lower limbs	***MYH7*** (Laing distal myopathy/ 160500)	XR /affected male sibling (MD13), Mother had a traffic accident and was non ambulatory. Motor ability cannot be evaluated.	Independent walking	Independent walking	Scoliosis	Mild restrictive lung disease	No	591	Myopathic	NE	NE	None	None
**MD13**[e]	M	1-5Y	Frequent falls, progressive distal muscle weakness of lower limbs, severe scoliosis	***MYH7*** (Laing distal myopathy/ 160500)	XR /Affected female sibling (MD12)	Independent walking	Independent walking	Scoliosis	Restrictive lung disease	No	1.204	Myopathic	Unspecific	NE	None	None
**MD14**	M	0-1M	Hypotonia and respiratory failure from perinatal period, AMC, delayed motor development, proximal muscle weakness, proximal joint contracture, finger flexion contracture	***TTN*** (Limb-girdle muscular dystrophy/ 608807)	Negative	Independent walking	Independent walking	Rigid spine	OSA	No	86	NE	Dystrophic change, decreased collagen6 staining	NE	Surveillance for cardiac, respiratory involvement, and scoliosis	None
**MD15**	M	1-5Y	Frequent falling, generalized hypotonia, progressive proximal muscle weakness, areflexia, Gowers' sign positive, waddling gait, toe walking	**No causative variant for muscular dystrophy was found.** A homozygous missense variant in the *FUS* gene known to cause amyotrophic lateral sclerosis, frontotemporal demential /608030)	Male sibling with proximal muscle weakness, onset 1.5 Y, Serum CPK 958 U/L, muscle pathology was similar to the patient's result, EM showed numerous abnormal mitochondria, deceased at age 9 Y due to respiratory failure	Independent walking	Independent walking	Lordosis	No	No	1.010	Myopathic	Vacuolated muscle fibers with granular eosinophilic and amphophilic materials. COX-negative fibers.	Mitochon-drial DNA sequencing for common mutation (A3243G, A8344G, T8993G)	None	None

**CM1**	F	0-1M	Polyhydramnios, preterm GA 32 weeks, very low birth weight 1300g, congenital hypotonia, with weakness, hyporeflexia, respiratory failure, no liver enlargement, normal liver function test	***GBE1*** *(*glycogen storage disease type IV/232500*)*	AR	Bed bound	Deceased at age 2M	No	Require mechanical ventilator	No	231	NE	Glycogen accumulation	Targeted gene panel for neuromas-cular disorders	None	None
**CM2**	M	0-1M	Congenital hypotonia with weakness, areflexia, low APGAR, high arched palate, lung atelectasis	***MTM1*** *(myotubular myopathy/ 310400)*	XR /Maternal history of spontaneous abortion	Bed bound	Bed bound	No	Require mechanical ventilator	No	50	NE	NE	SMA[c]	Muscle biopsy is not necessary.	None
**CM3**	F	1-12M	Delayed motor development, hypotonia, proximal muscle weakness, mild elongated face, high-arched palate, areflexia	***NEB*** *(Nemaline myopathy/ 256030)*	AR /Negative	Sitting alone	Sitting alone	No	No	No	78	NE	NE	SMA[c]	Muscle biopsy is not necessary.	None
**CM4**	M	1-12M	Severe scoliosis, myopathic face, proximal muscle weakness, history of malignant hyperthermia	***RYR1*** *biallelic (*central core disease /117000)	AR /Negative	Independent walking	Independent walking	Scoliosis	No	No	279	Myopathic	NE	*RYR1* limited mutation screening[d]	Muscle biopsy is not necessary. Avoidance of volatile anesthetics and depolarizing muscle relaxant	None
**CM5**	M	1-12M	Feeding difficulties, delayed motor development, myopathic face, high-arched palate, ptosis, ophthalmoplegia, proximal muscle weakness, hyporeflexia	***RYR1*** *Biallelic (RYR1-related centronuclear myopathy)*	AR /Negative	Independent walking	Independent walking	Lumbar lordosis	No	No	110	NE	Centronuclear	SMA[c]	Avoidance of volatile anesthetics and depolarizing muscle relaxant	None
**CM6**	M	0-1M	Hypotonia and respiratory impairment from perinatal period, feeding difficulties, myopathic face, high-arched palate, ptosis, ophthalmoplegia, proximal muscle weakness, areflexia	***RYR1*** *biallelic (congenital uniform fiber type I /117000)*	AR /Negative	Independent walking	Independent walking	No	OSA	No	57	Myopathic	Type 1 fiber predominance	SMA[c]	Avoidance of volatile anesthetics and depolarizing muscle relaxant	None

**CM7**	F	1-12M	Hypotonia, delay motor development, mild long face, high-arched palate	***RYR1*** *Monoallelic (RYR1-related congenital myopathy)*	AD /Negative	Independent walking	Independent walking	Lumbar lordosis	No	No	54	NE	NE		Muscle biopsy is not necessary. Avoidance of volatile anesthetics and depolarizing muscle relaxant	None
**CM8**	F	1-5Y	Waddling gait, proximal muscle weakness	***TTN*** *biallelic (VUS)*	Negative	Independent walking	Independent walking	Lumbar lordosis	No	No	193	NE	NE		None	None
**CM9**	M	0-1M	Hypotonia, respiratory impairment during perinatal period, proximal muscle weakness, myopathic face, high-arched palate, areflexia	***TTN*** (Limb-girdle muscular dystrophy/ 608807)	AR /Negative	Independent walking	Independent walking	No	Yes	Yes	57	NE	Centronuclear, fiber type disproportion	SMA[c], *RYR1* limited mutation screening[d]	None	None

Abbreviations: AD, autosomal dominant; ADHD, attention deficit hyperactive disorder; AMC, arthrogryposis multiplex congenita; AR; autosomal recessive, CK, creatine kinase (normal: < 190 U/L); CM, congenital myopathy; DMD, Duchenne muscular dystrophy; Edx, electrodiagnostic testing; EF, ejection fraction (from echocardiogram); GA, gestational age; F, female; GDD, global developmental delay; LVNC, left ventricular non compaction; M, male; MD, muscular dystrophy; NE, not examined; NIV, non-invasive ventilation; OSA, obstructive sleep apnea; PFT, pulmonary function test; RNS, repetitive nerve stimulation; SSCD, sarcolemma specific collagen deficiency, XR; X-linked recessive

In the patient-ID column, DMD, MD and CM represent patients clinically diagnosed with Duchenne muscular dystrophy, other muscular dystrophies and congenital myopathies, respectively.

[a] Describe respiratory and cardiac involvement manifested at the time of testing

[b] Multiplex ligation-dependent probe amplification (MLPA) for the *DMD* gene

[c] Multiplex ligation-dependent probe amplification (MLPA) of the *SMN1* gene

[d] *RYR1* limited mutation screening refers to PCR amplification of exons 10, 11, 39, 40, 41, 43, 44 and 45 of the *RYR1* gene

[e] Sibling relationship

Table S2. Molecular findings

Patient ID	ES	Gene	Reference sequence	Nucleotide change	Zygosity	Parental mutation status	Inheritance	Publication	ACMG classification[a]
**DMD1**	Singleton	*DMD*	NM_004006.3	c.515_516dupTC (p.Ile173SerfsTer36)	Hemizygous	Not confirmed	XR	This study	Pathogenic
**DMD2**	Singleton	*DMD*	NM_004006.3	c.5932delC (p.Arg1978ValfsTer5)	Hemizygous	Not confirmed	XR	This study	Pathogenic
**DMD3**	Trio	*DMD*	NM_004006.3	c.6986delA (p.Lys2329SerfsTer9)	Heterozygous	Maternal allele	XR	[PMID: 26911353]	Pathogenic
**DMD4**	Singleton	*DMD*	NM_004006.3	c.8086dupC (p.Leu2696ProfsTer14)	Heterozygous	Not confirmed	XR	[PMID: 31379145]	Pathogenic
**DMD5**	Singleton	*DMD*	NM_004006.3	c.8086delC (p.Leu2696TrpfsTer30)	Hemizygous	Not confirmed	XR	[PMID: 8840119]	Pathogenic
**DMD6**	Singleton	*DMD*	NM_004006.3	c.9649+5G>T	Heterozygous	Not confirmed	XR	[PMID: 20485447]	Likely pathogenic
**DMD7**	Trio	*DMD*	NM_004006.3	c.10097_10099delGAG (p.Gly3366delGAG)	Heterozygous	Maternal allele	XR	[PMID: 19937601]	Likely pathogenic
**DMD8**	Singleton	*DMD*	NM_004006.3	c.10108C>T (p.Arg3370Ter)	Hemizygous	Not confirmed	XR	[PMID: 25612904]	Pathogenic
**MD1**	Trio	*COL6A1*	NM_001848.2	c.850G>A (p.Gly284Arg)	Heterozygous	*De novo*	AD	[PMID: 34167565]	Likely pathogenic
**MD2**	Trio	*COL6A1*	NM_001848.2	c.868G>A (p.Gly290Arg)	Heterozygous	*De novo*	AD	[PMID: 30706156]	Pathogenic
**MD3**	Singleton	*COL6A1*	NM_001848.2	c.1056+1G>C	Heterozygous	Not confirmed	AD	[PMID: 25749816]	Pathogenic
**MD4**	Trio	*COLQ*	NM_005677.3	c.393+1G>A	Homozygous	Paternal and maternal allele	AR	[PMID 32978031]	Pathogenic
**MD5**	Singleton	*FHL1*	NM_001159702.2	c.377G>A (p.Cys126Tyr)	Heterozygous	Not confirmed	XR	[PMID: 24928078]	Likely pathogenic
**MD6**	Trio	*LAMA2*	NM_000426.4	c.283+1G>C<br>(no other pathogenic variants found)	Heterozygous	Paternal allele	AR	[PMID: 30055037]	Pathogenic
**MD7**	Singleton	*LAMA2*	NM_000426.4	c.2718delT (p.Phe906LeufsTer169)<br>c.7452-16T>G	Heterozygous<br>Heterozygous	Not confirmed	AR	This study<br>This study	Pathogenic<br>VUS
**MD8**	Trio	*LMNA*	NM_170707.4	c.116A>G (p.Asn39Ser)	Heterozygous	*De novo*	AD	[PMID: 26098624]	Pathogenic
**MD9**	Trio	*LMNA*	NM_170707.4	c.1072G>A (p.Glu358Lys)	Heterozygous	*De novo*	AD	[PMID: 29764566]	Pathogenic
**MD10**	Trio	*LMNA*	NM_170707.4	c.1153_1155delGAG (p.Glu385del)	Heterozygous	*De novo*	AD	This study	Pathogenic
**MD11**	Trio	*LMNA*	NM_170707.4	c.1381-2A>C	Heterozygous	*De novo*	AD	This study	Pathogenic
**MD12**	Trio	*MYH7*[b]	NM_000257.3	c.4807G>C (p.Ala1603Pro)	Heterozygous	Maternal allele	AD	[PMID: 29660325]	Likely pathogenic
**MD13**	Trio	*MYH7*[b]	NM_000257.3	c.4807G>C (p.Ala1603Pro)	Heterozygous	Maternal allele	AD	[PMID: 29660325]	Likely pathogenic
**MD14**	Trio	*TTN*	NM_001267550.2	c.38876-2A>C<br>c.2254C>A (p.Arg752Ser)	Compound<br>Heterozygous	Paternal allele<br>Maternal allele	AR	This study<br>This study	Pathogenic<br>VUS
**MD15**	Trio	*FUS*	NM_004960.2	c.616G>A (Gly206Ser)	Homozygous	Paternal and maternal allele	AR	[PMID: 20668259]	Likely pathogenic (Not etiologic)
**CM1**	Trio	*GBE1*	NM_000158.4	c.1561A>T (p.Lys521Ter)<br>c.1496T>A (p.Met499Lys)	Compound Heterozygous	Paternal allele<br>Maternal allele	AR	This study<br>This study	Pathogenic<br>Likely pathogenic
**CM2**	Singleton	*MTM1*	NM_000252.3	c.414_421dupGAGATACG (p.Ala141GlyfsTer17)	Hemizygous	Not confirmed	XR	This study	Likely pathogenic
**CM3**	Trio	*NEB*	NM_001164508.2	c.10612C>T (p.Arg3538Ter)<br>c.23967_23970delACCT (p.Pro7990SerfsTer154)	Compound Heterozygous	Paternal allele<br>Maternal allele	AR	[PMID: 27104957]<br>This study	Pathogenic<br>Pathogenic

**CM4**	Trio	*RYR1*	NM_000540.3	c.487C>T (p.Arg163Cys)<br>c.15089G>A (p.Arg5030His)	Compound<br>Heterozygous	Paternal allele<br>Maternal allele	AR	[PMID: 27586648]<br>This study	Pathogenic<br>Likely pathogenic
**CM5**	Trio	*RYR1*	NM_000540.3	c.11715G>C (p.Gln3905His)<br>c.9611C>T (p.Ala3204Val)	Compound<br>Heterozygous	Paternal allele<br>Maternal allele	AR	This study<br>[PMID: 28818389]	Likely pathogenic<br>Likely pathogenic
**CM6**	Trio	*RYR1*	NM_000540.3	c.11314C>T (p.Arg3772Trp)<br>c.10347+1G>A	Compound<br>Heterozygous	Paternal allele<br>Maternal allele	AR	[PMID: 24091937]<br>[PMID: 25957634]	Pathogenic<br>Pathogenic
**CM7**	Trio	*RYR1*	NM_000540.3	c.14558C>T (p.Thr4853Ile)	Heterozygous	*De novo*	AD	[PMID: 23394784]	Pathogenic
**CM8**	Trio	*TTN*	NM_001267550.2	c.3926C>T (p.Thr1309Ile)<br>c.28856T>C (p.Ile9619Thr)	Compound<br>Heterozygous	Paternal allele<br>Maternal allele	AR	This study<br>This study	VUS<br>VUS
**CM9**	Trio	*TTN*	NM_001267550.2	c.39109G>T (p.Glu13037Ter)<br>c.53348T>C (p.Leu17783Pro)	Compound<br>Heterozygous	Paternal allele<br>Maternal allele	AR	This study<br>This study	Pathogenic<br>VUS

Abbreviations: AD; autosomal dominant, AR; autosomal recessive, VUS; variant of uncertain significance, ES; exome sequencing, XR; X-linked recessive

[a] According to the American College of Medical Genetics and Genomics interpretation guidelines [PMID: 25741868]

[b] Sibling relationship

In the patient-ID column, DMD, MD and CM represent patients clinically diagnosed with Duchenne muscular dystrophy, other muscular dystrophies and congenital myopathies, respectively.

Table S3. Characteristics of the 18 novel variants

Gene	Reference sequence	Nucleotide change	SIFT	Polyphen-2	M-CAP	Mutation Taster	CADD score	dbSNP	gnomAD allele frequency	ACMG classification[a]	ClinVar Submitter Accession
DMD	NM_004006.3	c.515_516dupTC (p.Ile173SerfsTer36)	NA	NA	NA	Disease-causing	32	-	-	Pathogenic	SCV002546536
DMD	NM_004006.3	c.5932delC (p.Arg1978ValfsTer5)	NA	NA	NA	Disease-causing	34	-	-	Pathogenic	SCV002546547
GBE1	NM_000158.4	c.1561A>T (p.Lys521Ter)	NA	NA	NA	Disease-causing	42	rs773775991	0.0000041	Pathogenic	SCV002546548
GBE1	NM_000158.4	c.1496T>A (p.Met499Lys)	Damaging	Probably damaging	Damaging	Disease-causing	26.8	-	-	Likely pathogenic	SCV002546549
LAMA2	NM_000426.4	c.2718delT (p.Phe906LeufsTer169)	NA	NA	NA	Disease-causing	33	-	-	Pathogenic	SCV002546551
LAMA2	NM_000426.4	c.7452-16T>G	NA	NA	NA	Polymorphism	24	-	-	VUS	SCV002546552
LMNA	NM_170707.4	c.1153_1155delGAG (p.Glu385del)	NA	NA	NA	Disease-causing	22.5	rs1553265761	-	Pathogenic	SCV002546553
LMNA	NM_170707.4	c.1381-2A>C	NA	NA	NA	Disease-causing	35	-	-	Pathogenic	SCV002546554
MTM1	NM_000252.3	c.414_421dupGAGATACG (p.Ala141GlyfsTer17)	-	-	-	Disease-causing	23.8	-	-	Likely pathogenic	SCV002546537
NEB	NM_001164508.2	c.23967_23970delACCT (p.Pro7990SerfsTer154)	NA	NA	NA	Disease-causing	38	rs756384471	-	Pathogenic	SCV002546538
RYR1	NM_000540.3	c.15089G>A (p.Arg5030His)	Damaging	Probably damaging	Damaging	Disease-causing	32	rs747155223	0.0000517	Likely pathogenic	SCV002546539
RYR1	NM_000540.3	c.11715G>C (p.Gln3905His)	Damaging	Probably damaging	Damaging	Disease-causing	24.2	-	-	Likely pathogenic	SCV002546540
TTN	NM_001267550.2	c.38876-2A>C	NA	NA	NA	Disease-causing	33	rs1185989004	0.000021	Pathogenic	SCV002546541
TTN	NM_001267550.2	c.2254C>A (p.Arg752Ser)	Damaging	Possibly damaging	Damaging	Polymorphism	22.3	-	-	VUS	SCV002546542
TTN	NM_001267550.2	c.3926C>T (p.Thr1309Ile)	Tolerated	Probably damaging	Damaging	Disease-causing	22.6	-	-	VUS	SCV002546543
TTN	NM_001267550.2	c.28856T>C (p.Ile9619Thr)	Tolerated	Possibly damaging	Damaging	Disease-causing	22.7	rs530257812	0.00000401	VUS	SCV002546544
TTN	NM_001267550.2	c.39109G>T (p.Glu13037Ter)	NA	NA	NA	Disease-causing	58	-	-	Pathogenic	SCV002546545
TTN	NM_001267550.2	c.53348T>C (p.Leu17783Pro)	Damaging	Probably damaging	Damaging	Disease-causing	23.5	rs777712545	0.00000403	VUS	SCV002546546

SIFT, sorting intolerant from tolerant (http://sift.jcvi.org/); *Polyphen-2*, prediction of functional effects of human SNPs (http://genetics.bwh.harvard.edu/pph2/); *M-CAP*, Mendelian clinically applicable pathogenicity score (http://bejerano.stanford.edu/mcap/); *MutationTaser* (https://www.mutationtaster.org/); *CADD*, combined annotation dependent depletion (https://cadd.gs.washington.edu/*; recommended pathogenicity threshold >20); dbSNP* (https://www.ncbi.nlm.nih.gov/projects/SNP/); *gnomAD*, https://gnomad.broadinstitute.org/ NA, not applicable

[a] According to the American College of Medical Genetics and Genomics interpretation guidelines
